# Human Infection with Novel Spotted Fever Group *Rickettsia* Genotype, China, 2015

**DOI:** 10.3201/eid2212.160962

**Published:** 2016-12

**Authors:** Hao Li, Xiao-Ming Cui, Ning Cui, Zhen-Dong Yang, Jian-Gong Hu, Ya-Di Fan, Xue-Juan Fan, Lan Zhang, Pan-He Zhang, Wei Liu, Wu-Chun Cao

**Affiliations:** State Key Laboratory of Pathogen and Biosecurity, Beijing Institute of Microbiology and Epidemiology, Beijing, China (H. Li, X.-M. Cui, J.-G. Hu, Y.-D. Fan, P.-H. Zhang, W. Liu, W.-C. Cao);; The People's Army 154 Hospital, Xinyang, China (N. Cui, Z.-D. Yang, X.-J. Fan, L. Zhang)

**Keywords:** Spotted fever group rickettsiae, rickettsiosis, rickettsia, human infection, ticks, vector-borne infections, China

## Abstract

Only 4 species of spotted fever group rickettsiae have been detected in humans in China. However, phylogenetic analysis of samples from 5 ill patients in China indicated infection with a novel spotted fever group *Rickettsia*, designated *Rickettsia* sp. XY99. Clinical signs resembled those of severe fever with thrombocytopenia syndrome.

Spotted fever group (SFG) rickettsiae are globally distributed and mostly transmitted by ticks (*1*). Recently, emerging and reemerging SFG rickettsiae, such as *Rickettsia slovaca* (*2*), *R. aeschlimannii* (*3*), *R. massiliae* (*4*), *Candidatus* Rickettsia tarasevichiae (*5*,*6*), and *R. sibirica* subspecies *sibirica* BJ-90 (*7*), previously considered nonpathogenic, were found to infect humans. In addition, *R. parkeri* was confirmed to be pathogenic 65 years after its detection in ticks in 1939 (*8*).

In China, SFG rickettsioses are not listed as reportable diseases, and only 4 species of SFG rickettsiae (*R. heilongjiangensis*, *R. sibirica* subspecies *sibirica* BJ-90, *Candidatus* Rickettsia tarasevichiae, and *R. raoultii*) have been detected in human blood samples (*9*). In contrast, besides these pathogenic species, at least 4 other species of SFG rickettsiae (*R. sibirica* subspecies *mongolotimonae*, *R. monacensis*, *R. slovaca*, *Candidatus* Rickettsia hebeiii) have been detected in ticks, urging a wider search for cases in humans. We report infection of 5 patients with a novel SFG rickettsia in eastern central China.

## The Study

From March through November 2015, at the People’s Liberation Army 154 Hospital in Xinyang City, Henan Province, China, patients who were acutely symptomatic with fever and had a history of tick bites or animal contact within the past month were screened for SFG rickettsiae infection. At admission, EDTA-anticoagulated samples of peripheral blood were collected. DNA was extracted by using a QIAamp DNA Blood Mini Kit (QIAGEN, Germantown, MD, USA). Nested PCRs selective for outer membrane protein A (*omp*A) and citrate synthase (*glt*A) genes were concurrently performed to detect SFG rickettsial DNA (Technical Appendix Table 1). Positive amplicons were purified and then sequenced in both directions. Acute-phase (<7 days after illness onset) and convalescent-phase (>14 days after illness onset) serum samples were tested by indirect immunofluorescence assay (IFA) for IgG against *R. rickettsii* by using a commercially available IFA kit (Focus Diagnostics Inc., Cypress, CA, USA).

**Figure 1:**
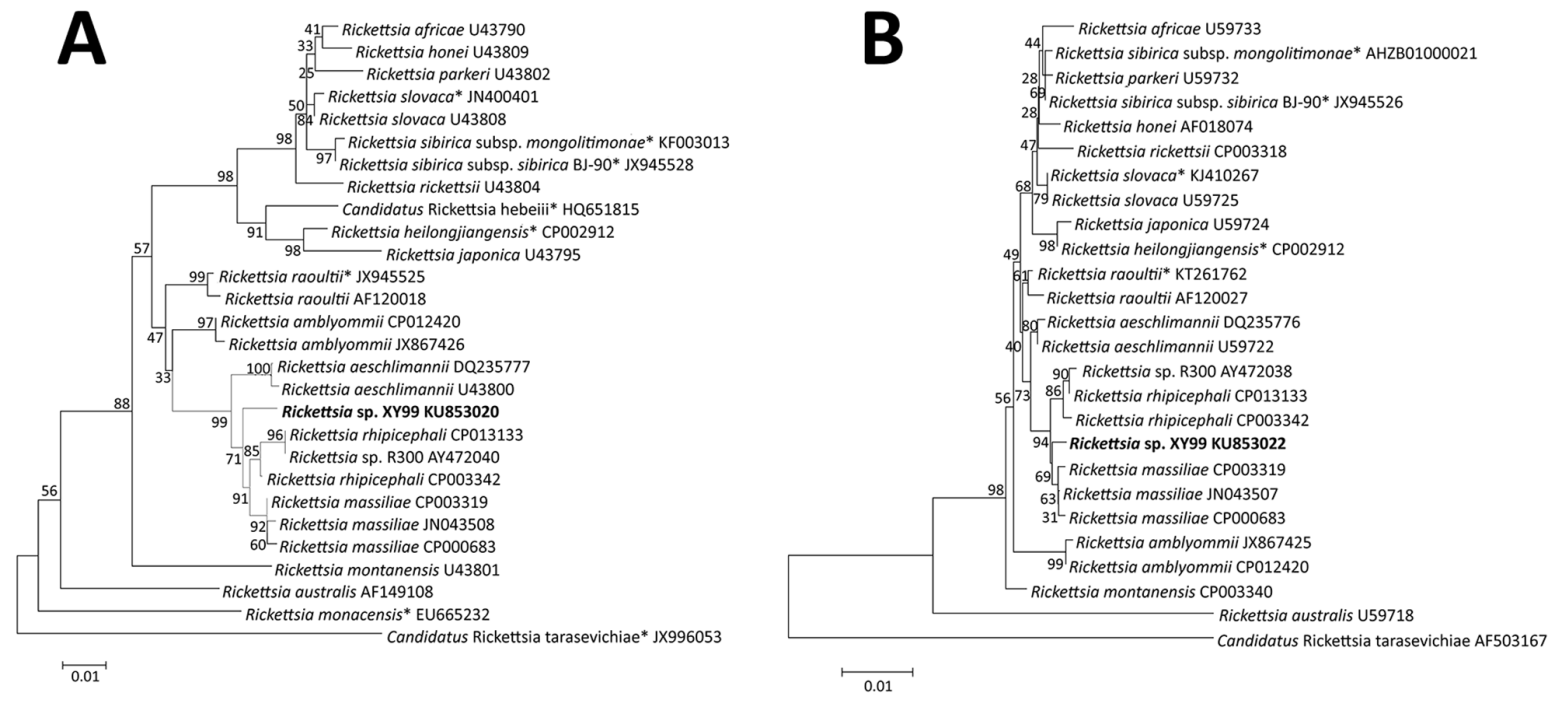
Phylogenetic analyses based on nucleotide sequences of the outer member protein A (307-bp) (A) and citrate synthase (1,150-bp) (B) genes of *Rickettsia*. Boldface indicates the newly discovered *Rickettsia* genotype (*Rickettsia* sp. XY99). Asterisks after taxon names indicate that the sequence of *Rickettsia* species was found in China. Neighbor-joining trees were conducted by using the maximum composite likelihood method by means of MEGA version 5.0 (http://www.megasoftware.net). Bootstrap analysis of 1,000 replicates was applied to assess the reliability of the reconstructed phylogenies. Scale bars indicate estimated evolutionary distance.

Positive amplification of *omp*A and *glt*A genes was found for 5 patients, and the obtained sequences for each of the 2 genes from all 5 patients were identical. Nucleotide sequence (350-bp) of *omp*A gene (GenBank accession no. KU853020) from each of the 5 patients showed 10-bp differences from that of *R. massiliae* strain AZT80 (GenBank accession no. CP003319) and 12-bp differences from that of *R. rhipicephali* strain HJ#5 (GenBank accession no. CP013133). Nucleotide sequences (1150-bp) of *glt*A gene (GenBank accession no. KU853022) from each of the 5 patients differed from that of *R. massiliae* strain AZT80 by 4 bp and from that of *R. rhipicephali* strain HJ#5 by 5 bp (Technical Appendix Table 2). According to phylogenetic analysis, the novel SFG rickettsiae genotype, here designated as *Rickettsia* sp. XY99, seems to represent a distinct lineage and could constitute a new species (Figure 1). For all 5 patients, seroconversion or a 4-fold increase of IgG against *R. rickettsii* was found between the acute- and convalescent-phase samples, and the patients were determined to have acute infection with SFG rickettsiae (Technical Appendix Table 3). Subsequent testing of the 5 patients for infection with severe fever with thrombocytopenia syndrome virus, *Anaplasma phagocytophilum*, “*A. capra*,” and *Babesia microti* by molecular (real-time PCR or nested PCR) and serologic tests (ELISA or IFA) produced no positive results.

**Figure 2:**
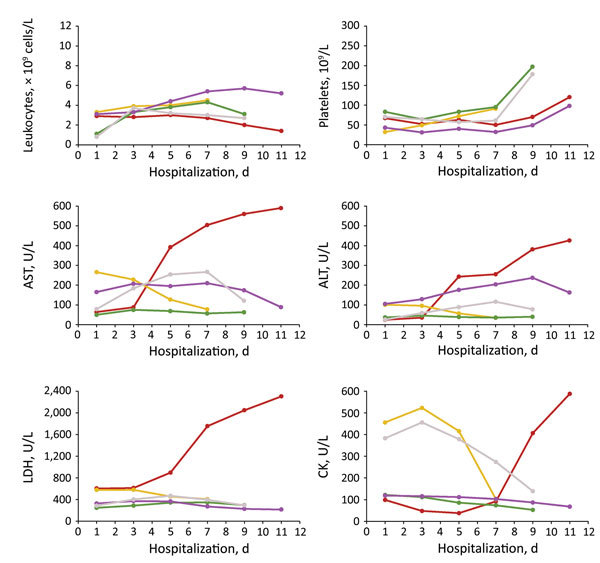
Dynamic changes of 6 laboratory parameters (with 2-day intervals) during hospitalization of 5 patients with *Rickettsia* sp. XY99 infection, China, 2015. Red, patient 1; yellow, patient 2; green, patient 3; purple, patient 4; gray, patient 5. ALT, alanine aminotransferase, reference range 0–40 U/L; AST, aspartate aminotransferase, reference range 0–40 U/L; CK, creatine kinase, reference range 25–200 U/L; LDH, lactate dehydrogenase, reference range 109–245 U/L; platelets, reference range 100–300 × 10^9^/L; leukocytes, reference range 4.0–10.5 × 10^9^ cells/L.

All 5 patients were farmers who resided in the villages of Xinyang City. Patient median age was 65 (range 62–80) years, and 3 were male (Table). Two patients had a history of tick exposure, and the other 3 had had contact with livestock. For all 5 patients, illness onset occurred June 20–July 10, 2015. The median time from illness onset to admission was 4 (range 3–6) days, and the median duration of hospitalization was 10 (range 8–12) days. All patients experienced fever (highest 38.4°C– 40.0°C), asthenia, anorexia, and nausea; 4 had cough, 3 vomiting, 2 myalgia, 1 headache, and 1 dizziness. Of note, all 5 patients had lymphadenopathy, but none had rash or eschar. At admission, all 5 patients had leukopenia, thrombocytopenia, and elevated hepatic aminotransferase levels; 4 had elevated lactate dehydrogenase levels, and 2 had elevated creatine kinase levels (Figure 2). Treatment included therapy with cefminox and cefoperazone; no doxycycline was used.

**Table:** Epidemiologic and clinical characteristics of 5 patients with *Rickettsia* sp. XY99 infection, China, 2015*

Characteristic	Patient no.				
	1	2	3	4	5
Age, y	65	64	66	80	62
Sex	M	F	F	M	M
History of tick bite	No	No	No	Yes	Yes
Time between tick bite and illness onset, d	NA	NA	NA	14	6
Time from onset to admission, d	3	6	5	4	4
Duration of hospitalization, d	12	8	9	12	10
Fever	Yes	Yes	Yes	Yes	Yes
Highest temperature, °C	40.0	39.5	38.7	38.4	39.1
Headache	Yes	No	No	No	No
Dizziness	No	Yes	No	No	No
Asthenia	Yes	Yes	Yes	Yes	Yes
Myalgia	Yes	Yes	No	No	No
Rash	No	No	No	No	No
Eschar	No	No	No	No	No
Lymphadenopathy	Yes	Yes	Yes	Yes	Yes
Anorexia	Yes	Yes	Yes	Yes	Yes
Nausea	Yes	Yes	Yes	Yes	Yes
Vomit	Yes	Yes	Yes	No	No
Cough	Yes	Yes	Yes	No	Yes
Pneumonia	Yes	No	Yes	No	Yes
Hydrothorax	Yes	No	Yes	No	No
Confusion	Yes	No	No	No	No
Meningeal irritation sign	Yes	No	No	No	No
Ecchymosis	Yes	No	No	No	No
Hemoptysis	No	No	No	No	Yes
Hematuria	Yes	Yes	No	No	No
*NA, not applicable.					

Complications included pneumonia (3 patients), hemorrhagic signs (3), hydrothorax (2), and neurologic syndromes (1). For 1 patient, severe complications progressively emerged 6 days after disease onset and included pneumonia and hydrothorax (Technical Appendix Figure), confusion, meningeal irritation sign, ecchymosis, and hematuria. Laboratory indicators were substantially more out of range 7 days after disease onset, indicative of severe multiorgan dysfunction (Figure 2). Treatment was ineffective, and the patient died 15 days after disease onset. The other 4 patients were discharged after 8–12 days’ hospitalization; at that time, all clinical signs and symptoms had resolved, but for certain patients, laboratory values remained out of reference range, suggesting slow recovery of organ dysfunction (Figure 2).

## Conclusions

Our detection of *Rickettsia* sp. XY99 DNA in blood samples collected during the acute period of illness (days 3–6 after onset) from 5 patients in the same region of China suggests that this organism was the etiologic agent of the infection. Seroconversion or a 4-fold increase in titers of IgG against *R. rickettsii* provided supportive evidence of SFG *Rickettsia* infection. Phylogenetic analysis indicated that *Rickettsia* sp. XY99 was a novel genotype of SFG rickettsiae.

In contrast to humans with *R. massiliae* infection and many other SFG rickettsioses reported previously (*4*,*10*), none of the 5 patients infected with *Rickettsia* sp. XY99 had rash or eschar, and only 1 had headache. In recent years, the concept of the classic triad of fever, rash, and headache suggesting infection with SFG rickettsiae has been increasingly challenged. Several emerging SFG rickettsiae species, such as *R. slovaca* (*2*), *R. raoultii* (*11*), *R. africae* (*12*), and *R. helvetica* (*13*), can infect humans, but such infections lack these traditional features, which were also lacking in the cases reported here. Therefore, absence of rash and tick-bite history should not exclude suspicion of SFG rickettsiae infection.

Similar to *R. slovaca* and *R. raoultii* infections, which can be associated with tickborne lymphadenopathy and *Dermacentor*-borne necrosis-erythema-lymphadenopathy (*14*), lymphadenopathy was also observed in all 5 patients with *Rickettsia* sp. XY99 infection. Thus, lymphadenopathy might be a typical sign useful for clinical diagnosis of *Rickettsia* sp. XY99 infection. All 5 patients had gastrointestinal syndromes, indicating potential tissue lesions or vascular injury of the gastrointestinal tract. The hydrothorax and multiple hemorrhagic signs in 4 patients is possibly suggestive of vascular invasion or damage caused by this novel *Rickettsia* species.

Confirmation of the novel *Rickettsia* genotype was achieved only by sequencing the *omp*A and *glt*A genes. Although differences based on 2 gene segments support its designation as a novel species, isolation efforts and characterization of all 5 genes (*rrs*, *glt*A, *omp*A, *omp*B, and *gene*D) are warranted, according to the guidelines for classification of a new *Rickettsia* species (*15*).

Physicians in this area of China should be aware of human infections with *Rickettsia* sp. XY99. It should be included in differential diagnoses with severe fever with thrombocytopenia syndrome, which causes similar clinical illness and is also endemic to the same area in eastern central China.

Technical Appendix Additional materials and methods used in study of Human infection with novel spotted fever group *Rickettsia* genotype, China, 2015.
