# Supplementary material for: Human Infection with Novel Spotted Fever Group Rickettsia Genotype, China, 2015
Source: Emerg Infect Dis. 2016 Dec;22(12):2153–6. doi: 10.3201/eid2212.160962 (PMC5189162; doi:10.3201/eid2212.160962)
Supplement: Technical Appendix — Additional materials and methods used in study of Human infection with novel spotted fever group Rickettsia genotype, China, 2015. [file 16-0962-Techapp-s1.pdf]

Article DOI: http://dx.doi.org/10.3201/eid2212.160962

# Human Infection with Novel Spotted Fever Group *Rickettsia* Genotype, China, 2015

## Technical Appendix

## Materials

### Molecular detection of rickettsial infection

DNA was extracted from blood specimens collected at admission with the use of the QIAamp Blood Mini Kit (Qiagen) according to manufacturer's instructions. Nested PCR assays targeting the *ompA* and *gltA* genes were concurrently performed to detect the presence of SFG rickettsial DNA. Nucleotide sequences of the primers were shown in Technical Appendix Table 1.

## Results

Serologic test results for 5 patients with *Rickettsia* sp. XY99 infection were shown in Technical Appendix Table 2.

Patient 1 developed pneumonia and hydrothorax as shown in the Technical Appendix Figure.

### References

1. Regnery RL, Spruill CL, Plikaytis BD. Genotypic identification of rickettsiae and estimation of intraspecies sequence divergence for portions of two rickettsial genes. J Bacteriol. 1991;173:1576–89.

2. Jia N, Zheng YC, Jiang JF, Ma L, Cao WC. Human infection with *Candidatus Rickettsia tarasevichiae*. N Engl J Med. 2013;369:1178–80. http://dx.doi.org/10.1056/NEJMc1303004

3. Mediannikov OY, Sidelnikov Y, Ivanov L, Mokretsova E, Fournier P-E, Tarasevich I, et al. Acute tick-borne rickettsiosis caused by *Rickettsia heilongjiangensis* in Russian Far East. Emerg Infect Dis. 2004;10:810–7. http://dx.doi.org/10.3201/eid1005.030437

4. Zhan L, He J, Saren GW, Wu XM, Wang JB, Zhao QM, et al. Investigation on *Anaplasma phagocytophilum* infection in rodents from forest areas in northeastern China. Chin J Epidemiol. 2007;28:157–9.

5. Li H, Zheng YC, Ma L, Jia N, Jiang BG, Jiang RR, et al. Human infection with a novel tick-borne *Anaplasma species* in China: a surveillance study. Lancet. 2015;15:663–70. http://dx.doi.org/10.1016/S1473-3099(15)70051-4

6. Persing DH, Mathiesen D, Marshall WF, Telford SR, Spielman A, Thomford JW, et al. Detection of *Babesia microti* by polymerase chain reaction. J Clin Microbiol. 1992;30:2097–103.

7. Liu W, Lu QB, Cui N, Li H, Wang LY, Liu K, et al. Case-fatality ratio and effectiveness of ribavirin therapy among hospitalized patients in China who had severe fever with thrombocytopenia syndrome. Clin Infect Dis. 2013;57:1292–9. http://dx.doi.org/10.1093/cid/cit530

Publisher: CDC; Journal: Emerging Infectious Diseases
Article Type: Dispatch; Volume: 22; Issue: 12; Year: 2016; Article ID: 16-0962
DOI: 10.3201/eid2212.160962; TOC Head: Dispatch

**Technical Appendix Table 1.** Nucleotide sequences of the primers used in the study

| Organism | Target gene | Primer | Sequence (5'-3') | Reference |
|---|---|---|---|---|
| Spotted fever group *Rickettsia* | *omp*A* | Rr190.70p | ATGGCGAATATTTCTCCAAAA | (*1*) |
| | | Rr190.602n | AGTGCAGCATTCGCTCCCCCT | |
| | | 190.70-38s1 | AAAACCGCTTTATTCACC | (*2*) |
| | | 190.602-384r1 | GGCAACAAGTTACCTCCT | |
| | *glt*A† | CS1d | ATGACTAATGGCAATAATAA | (*3*) |
| | | CSEndr | CTTATACTCTCTATGTACA | |
| | | RpCS877p | GGGGACCTGCTCACGGCGG | |
| | | RpCS1258n | ATTGCAAAAAGTACAGTGAACA | |
| *Anaplasma phagocytophilum* | *glt*A | W1 | TGTTTTGGAGTGTGGAGAC | (*4*) |
| | | W1 | GGTGAACCAATCTCAGCAA | |
| | | N1 | ATATAGAAAATCTGATCGG | |
| | | N2 | CTCTAAGTTTGCGTCAGC | |
| *"A. capra"* | *glt*A | Outer-f | GCGATTTTAGAGTGYGGAGATTG | (*5*) |
| | | Outer-r | TACAATACCGGAGTAAAAGTCAA | |
| | | Inner-f | GGGTTCMTGTCYACTGCTGCGTG | |
| | | Inner-r | TTGGATCGTARTTCTTGTAGACC | |
| *Babesia microti* | 18S rRNA | Bab1 | CTTAGTATAAGCTTTTATACAGC | (*6*) |
| | | Bab4 | ATAGGTCAGAAACTTGAATGATACA | |
| | | Bab2 | GTTATAGTTTATTTGATGTTCGTTT | |
| | | Bab3 | AAGCCATGCGATTCGCTAAT | |
| severe fever with thrombocytopenia syndrome virus | S-segment | Forward | TTCACAGCAGCATGGAGAGG | (*7*) |
| | | Reverse | GATGCCTTCACCAAGACTATCAATG | |
| | | Probe | FAM-AACTTCTGTCTTGCTGGCTCCGC-TAMRA | |

*Nucleotide positions of the four primers are 1-21, 513-533, 52-69, and 381-398, referring to the *omp*A sequence of *Rickettsia heilongjiangensis* (GenBank accession number AF179362).

†Nucleotide positions of the four primers are 1-20, 1272-1290, 797-815, and 1157-1178, referring to the *omp*A sequence of *R. helvetica* (GenBank accession number KU310588).

Publisher: CDC; Journal: Emerging Infectious Diseases
Article Type: Dispatch; Volume: 22; Issue: 12; Year: 2016; Article ID: 16-0962
DOI: 10.3201/eid2212.160962; TOC Head: Dispatch

**Technical Appendix Table 2.** Sequence similarity of *omp*A and *glt*A genes between *Rickettsia* sp. XY99 and other *Rickettsia* strains

| | | | | *omp*A | | *glt*A | |
|---|---|---|---|---|---|---|---|
| Species | Strain | Country | Host | GenBank accession number | Similarity | GenBank accession number | Similarity |
| *R. massiliae* | AZT80 | USA | Human | CP003319 | 340/350 | CP003319 | 1146/1150 |
| | MTU5 | Cyprus | Human | CP000683 | 339/350 | CP003319 | 1146/1150 |
| | 56m | Italy | Human | KJ663747 | 340/350 | - | - |
| | I20 | Israel | Tick | KJ187077 | 339/350 | - | - |
| | GL041 | Guinea | Tick | JN043508 | 339/350 | - | - |
| *R. rhipicephali* | 3-7-6 | - | - | CP003342 | 340/350 | CP003342 | 1145/1150 |
| | HJ#5 | Brazil | Tick | CP013133 | 338/350 | CP013133 | 1145/1150 |
| | Do290 | USA | Tick | EU109176 | 339/350 | - | - |
| *Rickettsia* sp. R300 | R300 | Brazil | Tick | AY472040 | 338/350 | AY472038 | 1144/1150 |
| *R. aeschlimannii* | MC16 | Morocco | Tick | U43800 | 335/350 | U59722 | 1142/1150 |
| | Stavropol | Russia | Tick | DQ235777 | 336/350 | DQ235776 | 1141/1150 |
| *R. amblyommii* | Ac37 | Brazil | Tick | CP012420 | 327/350 | CP012420 | 1134/1150 |

**Technical Appendix Table 3.** Serologic test results for 5 patients with *Rickettsia* sp. XY99 infection*

| | | | Days after onset | | IFA* | |
|---|---|---|---|---|---|---|
| Patient No. | Age | Sex | AP | CP | AP | CP |
| Patient 1 | 65 | M | 4 | 15 | <64 | 256 |
| Patient 2 | 64 | F | 7 | 14 | 64 | 256 |
| Patient 3 | 66 | F | 6 | 14 | <64 | 128 |
| Patient 4 | 80 | M | 5 | 16 | <64 | 128 |
| Patient 5 | 62 | M | 5 | 14 | <64 | 64 |

*Performed by detection of IgG against *R. rickettsia*. AP, acute phase; CP, convalescent phase; IFA, indirect immunofluorescence assay.

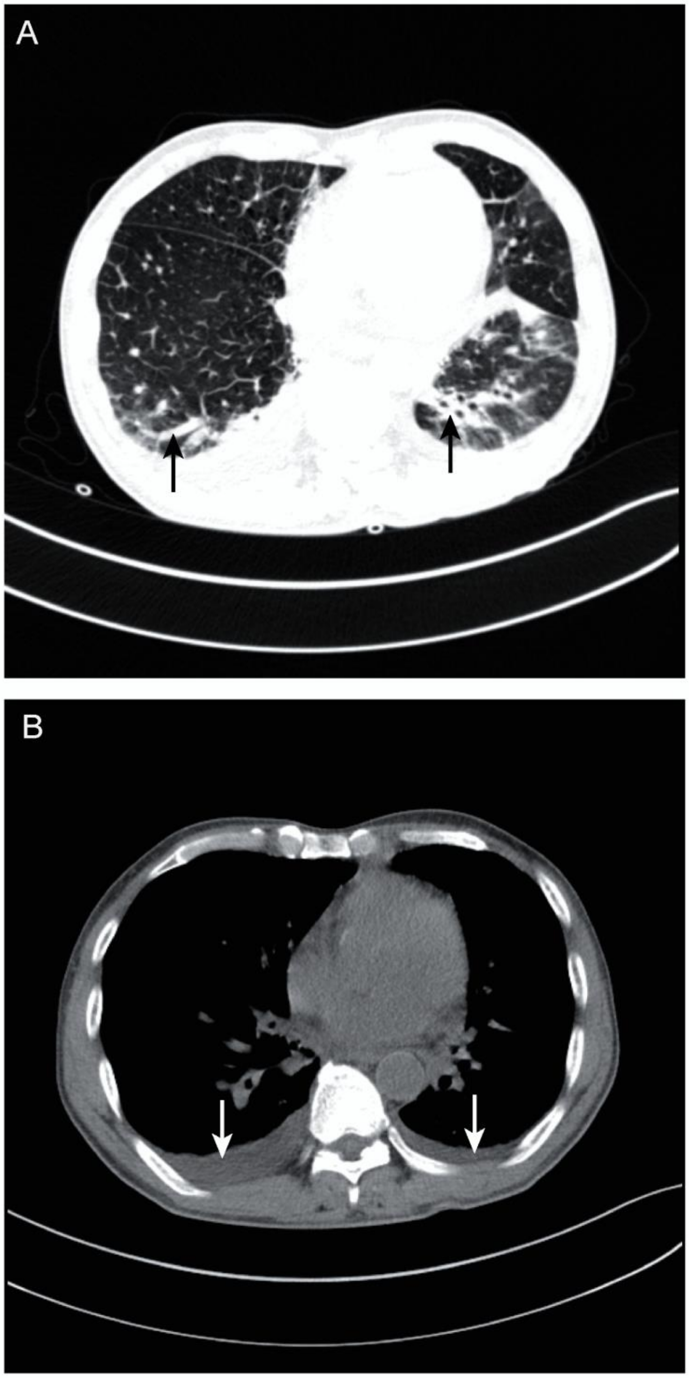


**Technical Appendix Figure.** The presence of pneumonia and hydrothorax in Patient 1 revealed by computerized tomography. Panel A showed exudative lesions on both lower lung; Panel B showed serous fluid on both pleural cavities.
